# Validation of *Tuba1a* as Appropriate Internal Control for Normalization of Gene Expression Analysis during Mouse Lung Development

**DOI:** 10.3390/ijms16034492

**Published:** 2015-02-25

**Authors:** Aditi Mehta, Stephanie Dobersch, Reinhard H. Dammann, Saverio Bellusci, Olga N. Ilinskaya, Thomas Braun, Guillermo Barreto

**Affiliations:** 1 LOEWE Research Group Lung Cancer Epigenetic, Max-Planck-Institute for Heart and Lung Research, Parkstraße 1, 61231 Bad Nauheim, Germany; E-Mails: aditi.mehta@mpi-bn.mpg.de (A.M.); stephanie.dobersch@mpi-bn.mpg.de (S.D.); 2 Universities of Giessen and Marburg Lung Center (UGMLC), Aulweg 130, 35392 Giessen, Germany; E-Mails: reinhard.dammann@gen.bio.uni-giessen.de (R.H.D.); Saverio.Bellusci@innere.med.uni-giessen.de (S.B.); Thomas.Braun@mpi-bn.mpg.de (T.B.); 3 German Center of Lung Research (DZL), Aulweg 130, 35392 Giessen, Germany; 4 Institute for Genetics, Justus-Liebig-University, Heinrich-Buff-Ring 58, 35392 Giessen, Germany; 5 Chair for Lung Matrix Remodeling, Excellence Cluster Cardio Pulmonary System, Aulweg 130, 35392 Giessen, Germany; 6 Institute of Fundamental Medicine and Biology, Kazan (Volga Region) Federal University, 18 Kremlyovskaya St, 420008 Kazan, Russian Federation; E-Mail: Olga.Ilinskaya@ksu.ru; 7 Department of Cardiac Development and Remodeling, Max-Planck-Institute for Heart and Lung Research, Parkstraße 1, 61231 Bad Nauheim, Germany

**Keywords:** mouse lung development, reference gene, qRT-PCR, geNorm, alpha 1A tubulin, *Tuba1a*

## Abstract

The expression ratio between the analysed gene and an internal control gene is the most widely used normalization method for quantitative RT-PCR (qRT-PCR) expression analysis. The ideal reference gene for a specific experiment is the one whose expression is not affected by the different experimental conditions tested. In this study, we validate the applicability of five commonly used reference genes during different stages of mouse lung development. The stability of expression of five different reference genes (*Tuba1a*,* Actb Gapdh*,* Rn18S* and* Hist4h4*) was calculated within five experimental groups using the statistical algorithm of geNorm software. Overall, *Tuba1a* showed the least variability in expression among the different stages of lung development, while *Hist4h4* and *Rn18S* showed the maximum variability in their expression. Expression analysis of two lung specific markers, surfactant protein C (*SftpC*) and Clara cell-specific 10 kDA protein (*Scgb1a1*), normalized to each of the five reference genes tested here, confirmed our results and showed that incorrect reference gene choice can lead to artefacts. Moreover, a combination of two internal controls for normalization of expression analysis during lung development will increase the accuracy and reliability of results.

## 1. Introduction

Quantification of transcript levels constitutes one of the crucial aspects of characterizing samples in molecular biology. Techniques to analyse gene expression include reverse transcription followed by polymerase chain reaction (RT-PCR), Northern Blotting,* in situ* hybridization and microarray-based expression analysis. Due to several reasons, use of quantitative PCR to amplify cDNA reverse transcribed from RNA (qRT-PCR) became a routine tool and is the most common technique. Quantitative RT-PCR is cost-efficient and uncomplicated to perform, allowing rapid throughput. In addition, qRT-PCR provides the specificity that is required for accurate and reliable quantification results. Finally, qRT-PCR is highly sensitive and permits the detection of rare transcripts and small changes in gene expression. Even a single cell has been used for qRT-PCR based expression analysis [1].

Our study deals with the relative quantification of gene expression by qRT-PCR. One of the critical aspects of relative gene expression analysis is normalization. Several strategies have been proposed to normalize the analysis of transcript levels, including similar starting material based on sample size or tissue volume, cell numbers, amount of total RNA or genomic DNA, use of external RNA as a “spike-in” standard [2] and comparison with an endogenous reference gene transcript [3,4]. A combination of two or more of these normalization strategies is frequently used to increase the reliability of the data. However, the expression ratio between the analysed gene and an internal control gene is the most widely used normalization method. Importantly, correct choice of the reference gene is critical to this normalization method, since incorrect reference gene choice can lead to artefacts [5]. The ideal reference gene for a specific experiment is the one whose expression is not affected by the different experimental conditions tested. Thus it is highly recommended to validate the selected reference gene for each experimental set up. In this study, we addressed this issue, focusing on mouse lung development.

The mouse lung originates from the anterior endoderm and forms during five phases of lung development: embryonic (9.5 to 12.5 days post coitum; E9.5–E12.5), pseudoglandular (E12.5–E16.5), canalicular (E16.5–E17.5), saccular (E17.5 to 5 days after birth; E17.5–P5) and alveolar (P5–P28) [6,7,8]. At the end of the embryonic phase, primary and secondary lung bud formation has taken place and the embryonic lung consists of one left lobe and four right lobes. From E10.5 to E16.5, the epithelium undergoes branching morphogenesis to form the respiratory (bronchial) tree. In parallel to branching morphogenesis, the primitive lung epithelium differentiates to several specialized cell types. However, most of the differentiation occurs in the canalicular and saccular phases (E16.5–P5). We have selected five of the most commonly used genes as reference for normalization of expression analysis during mouse lung development in previous reports [9] (Table 1). These genes have been involved in different cellular processes, alpha 1a tubulin (*Tuba1a*) and beta actin (*Actb*) are both required for cellular cytoskeleton [10]; glyceraldehyde 3 phosphate dehydrogenase (*Gapdh*) is a key enzyme in the control of glycolysis [5,11]; histone H4 (*Hist4h4*) is central to nucleosome formation [12] and 18S ribosomal RNA (*Rn18S*) is a structural component of eukaryotic ribosomes [13]. The objective of this study was to determine the validity of these genes as endogenous controls for the normalization of qRT-PCR-based expression analysis during the different phases of mouse lung development.

Our work is the first exhaustive study to validate internal controls for expression analysis through lung development. The relevance of our work was demonstrated by changing the results of qRT-PCR expression analysis for two epithelial markers during lung development when inappropriate reference genes were used for normalization. Proper verification and subsequent selection of suitable endogenous controls for a specific experimental setup will prevent inadequate quantification. The stability of expression of an internal control is required for accurate and reliable normalization in expression analysis experiments.

## 2. Results

### 2.1. Design of Primer Pairs for qRT-PCR Expression Analysis of Reference Genes that Are Widely Used in Mouse Lung Development

**Figure 1:**
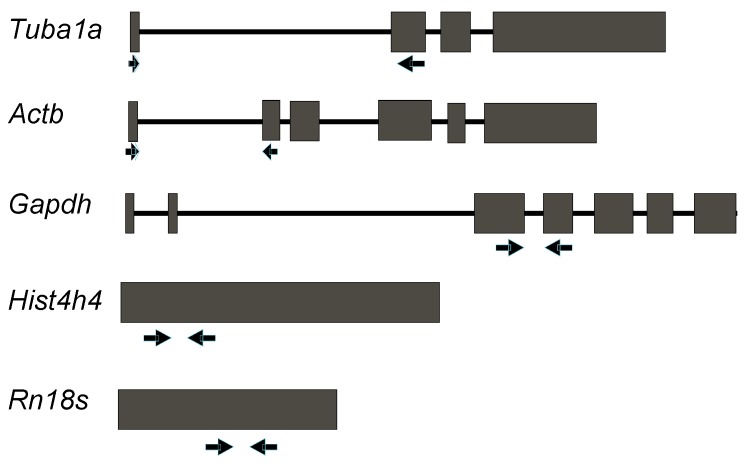
Gene structure of markers commonly used as internal controls for expression analysis during mouse lung development. Schematic representation of the gene structure of commonly used reference genes for expression analysis. Exons are represented as grey boxes and introns as black lines, while primer binding sites are shown with arrows.

Primer pairs for qRT-PCR-based expression analysis of the genes chosen for the present study were designed using the Geneious software [14] (Table 1)*.* Primer pairs for *Tuba1a*, *Actb* and *Gapdh* were spanning an intron (Figure 1) to avoid artefacts produced by leftovers of genomic DNA or precursor mRNA during quantitative PCR amplification of cDNA after reverse transcription from RNA. The primer pairs for *Hist4h4* and *Rn18S* were located in one exon due to their gene structure. A nucleotide BLAST (Basic Local Alignment Search Tool) search of the mouse RefSeq RNA database using the sequence of the designed primers as query revealed that the selected primer pairs specifically bind to their respective target transcripts and amplify only a single amplicon of the expected length, as supported by NCBI *E*-values lower than 0.05 (Table 2). In addition, the primer pairs were designed in order to amplify any known transcript variants or isoforms of their respective target. Interestingly, the analysis for *Actb* revealed that the mRNA sequence shares 91% homology with *Lrrc58* (leucine rich repeat containing 58). Thus, primer pairs were designed such that only the specific amplicon of *Actb* was obtained. Further, using the tool jPCR [15] and the Sigma Aldrich Oligo Evaluator online tool, it was confirmed that the primer pairs did not form primer dimers, had a similar annealing temperature, amplified amplicons of comparable length (between 151 and 312 base pairs) and did not form secondary structures for all the genes analysed in this study (Table 2).

**Table 1:** Internal control genes evaluated in this study.

Gene Symbol	Accession	Name	Function	Localization	Pseudogenes ^†^
*Tuba1a*	NM_011653	tubulin, alpha 1A	Essential for structure and kinetics of cytoskeleton	Chr 15 (15 F1; 15 55.29 cM)	3
*Actb*	NM_007393	actin, beta	Essential for structure and kinetics of cytoskeleton	Chr 5 (5 G2; 5 81.8 cM)	39
*Gapdh*	NM_008084	glyceraldehyde-3-phosphate dehydrogenase	Important enzyme of glycolytic pathway	Chr 6 (6 F2; 6 59.32 cM)	309
*Hist4h4*	NM_175652	histone cluster 4, H4	Structural component of nucleosome	Chr 6 (6 G1; 6)	0
*Rn18S*	NR_003278	18S ribosomal RNA	Ribosomal subunits	Chr 6 (6; 17)	1

**^†^** Related pseudogene(s) reported on the NCBI database.

**Table 2:** Primers for internal control genes.

Gene Symbol	Forward Primer (5'–3')	*T*m (°C)	*E* Value	Reverse Primer (5'–3')	*T*m (°C)	*E* Value	Primer Dimer	Secondary Structure •	Intron Spanning	Amplicon Size	Slope (R > 0.99) ^†^	Amplification Efficiency (%) ^‡^
*Tuba1a*	CCGCGAAGCAGCAACCAT	61.43	0.012	CCAGGTCTACGAACACTGCC	60.39	0.001	No	None	Yes	227	−3.3711	97.36
*Actb*	ACACCCGCCACCAGTTC	64.78	0.032	TACAGCCCGGGGAGCAT	62.21	0.032	No	Very weak	Yes	110	−3.3797	97.64
*Gapdh*	TGAGTATGTCGTGGAGTCTAC	56.33	4 × 10^−4^	TGGACTGTGGTCATGAGCC	59.32	0.004	No	None	Yes	261	−3.3904	97.22
*HistH4*	ATGTCAGGACGAGGAAAAGGC	60.34	4 × 10^−4^	TTAGCCGCCGAAGCCGTAC	62.70	0.004	No	None	No	312	−3.4822	99.17
*Rn18S*	GTAACCCGTTGAACCCCATT	58.1	0.013	CCATCCAATCGGTAGTAGCG	57.93	0.013		None	No	151	−3.4239	95.91

**^†^** Slope of the linear regression line along the coefficient of regression, R, analysed by triplicate qRT-PCR reactions; **^‡^** Amplification efficiency calculated using the formula, *E* = 10^(−1/Slope)^ − 1; • Based on ∆*G* calculations at 45 °C.

### 2.2. Characterization of Primer Pairs by Determining the Amplification Efficiency and through Dissociation Curve Analysis

**Figure 2:**
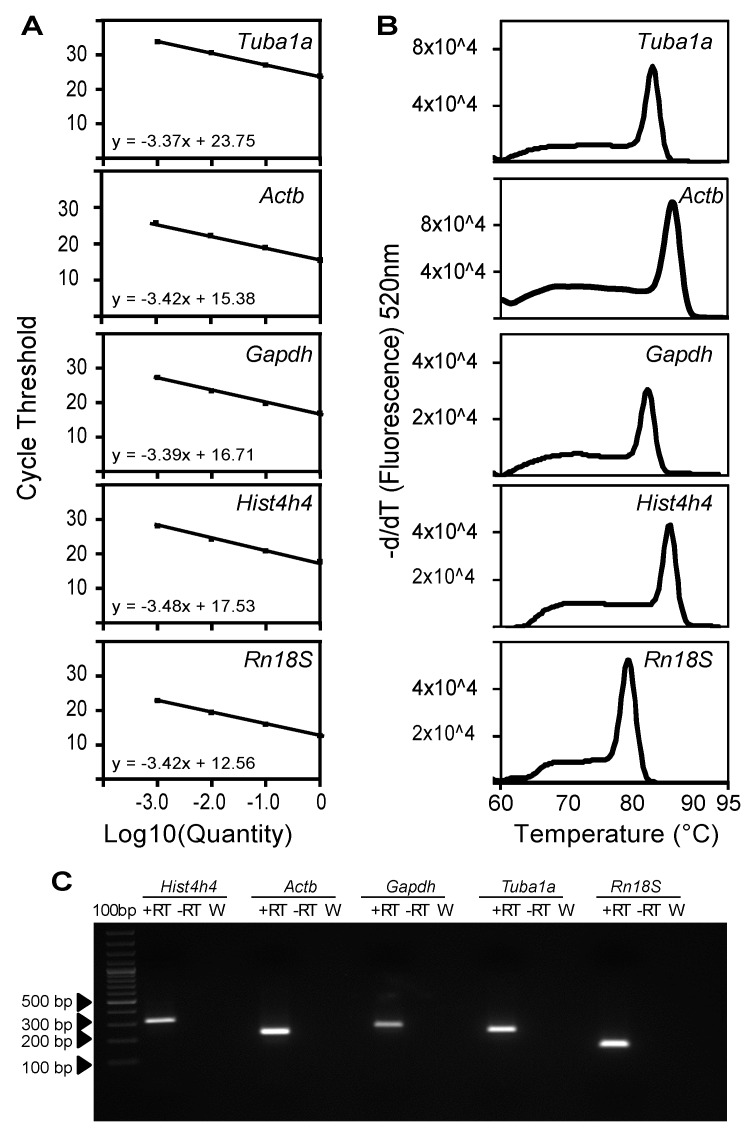
Characterization of primer pairs designed for expression analysis of the internal control during mouse lung development. (**A**) Amplification efficiency for each primer pair was calculated using 10-fold serial dilutions of a cDNA template. Primer amplification efficiency was assessed by plotting the cycle threshold (*C*_T_) value for each concentration against the logarithm (base 10) of the fold dilution (log_10_ (Quantity)). Efficiency was calculated using the slope of the linear function; (**B**) Dissociation curve analysis of primer specific products was performed by constantly monitoring the fluorescence with increasing temperatures from 60 to 95 °C. Melt curves were generated by plotting the negative first derivative of the fluorescence (−d/d*T* (Fluorescence) 520 nm)* versus* temperature (degree Celsius, °C); (**C**) Agarose gel electrophoresis after qRT-PCR indicates a specific product for each of the primer pairs. +RT, template is cDNA; −RT, template is RNA without reverse transcriptase, W, water control.

**Figure 3:**
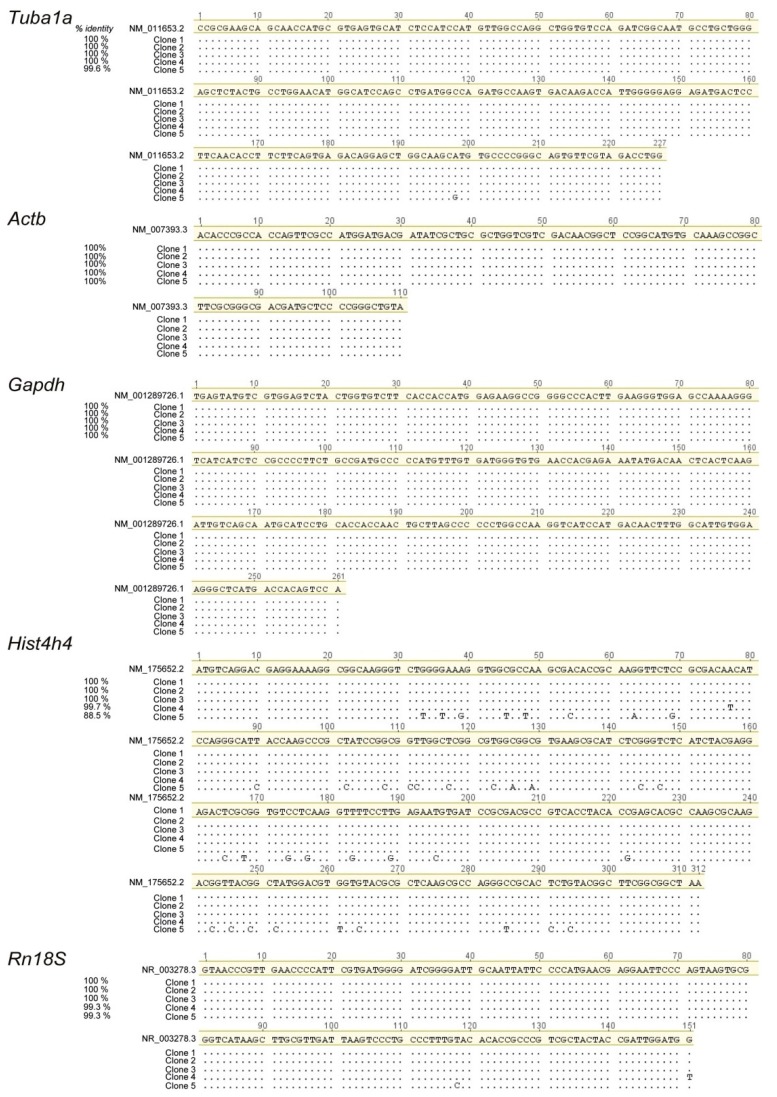
Sequence identity of the PCR products for the five reference genes evaluated in this study. Five plasmid clones (Clone 1–5) containing the PCR products of each of the reference genes evaluated in this study were compared against the reference sequence available at NCBI (Highlighted in yellow). Dots represent bases identical to the reference sequence while mismatches are indicated. Percentage of identity (% identity) is indicated for each clone.

Optimal primer pair design achieves doubling of the specific PCR product after each cycle of the PCR reaction during the exponential phase of amplification. In this ideal situation, the amplification efficiency of the primer pair would be 100% and the qRT-PCR results can be analysed using the 2^(−∆∆*C*T)^ method [16]. We decided to determine the amplification efficiency of the primer pairs that we designed for each one of the genes analysed in this study (Figure 2). Therefore we have performed qPCRs using the different primer pairs and, as a template, tenfold serial dilutions of a cDNA mixture of known concentration. The results of these qPCRs were used to elaborate a standard curve for each one of the primer pairs by plotting the template quantity (log_10_; *x*-axis) against the threshold cycle (*C*_T_; *y*-axis) (Figure 2A). The resulting standard curves can be described by the function *y* = m*x* + c, where m is the slope of the line, c is the *y*-axis intercept and *x* is the independent variable of the function *y* = *f*(*x*) The amplification efficiency (*E*) of each primer pair was calculated using the slope of the standard curve and the formula, *E* = (10^(−1/Slope)^ − 1) × 100% [17] (Table 2). Four of the five primer pairs analysed in this study had amplification efficiencies above 97%. Only the primer pair for *Rn18S* showed an amplification efficiency of 95.9%. However, the amplification efficiency of all the primer pairs analysed here was within the acceptable range of 100% ± 10% [18]. In addition, the dissociation curve analysis of the different qRT-PCR products showed a single peak for each one of the genes (Figure 2B), confirming the absence of non-specific PCR products, primer dimers or secondary structures [19]. Further, agarose gel electrophoresis after qRT-PCR demonstrated a single band of the expected size only in the sample containing the template, while no product was obtained in the negative controls without reverse transcriptase (−RT) or with water (Figure 2C), confirming again the specificity of the primer pairs and the absence of genomic DNA contamination. Lastly, sequencing of the PCR products obtained from each of the reactions confirmed the identity of the amplicons and the specificity of the analysis (Figure 3). For *Tuba1a*,* Actb*,* Gapdh* and *Rn18S*, all the five clones showed specificity to the intended product. However, for *Hist4h4*, Clone 5 showed only 88% homology to the target. This can be explained by the fact that the histone cluster genes are highly conserved and all of them give rise to an identical Histone 4 (H4) protein. Although the designed primers were targeting *Hist4h4*, the primers also recognized *Hist2h4*, despite considerable mismatch, resulting in a product of identical size. Clone 5 was highly similar to *Hist2h4*, a Histone 4 variant that is encoded by Histone Cluster 2, which shows only 86% homology to *HistH4h.*

### 2.3. Tuba1a Is the Least Variable Endogenous Reference for the Normalization of qRT-PCR-Based Expression Analysis during Mouse Lung Development

**Figure 4:**
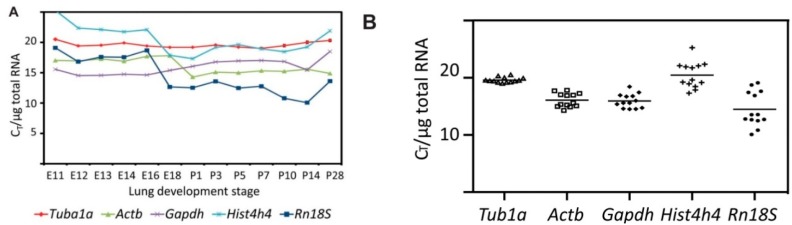
Expression analysis of the internal control genes evaluated in this study during different phases of lung development. (**A**) Variable cycle threshold (*C*_T_) for the different reference genes over mouse lung development. *C*_T_ values were determined by qRT-PCR in triplicates for biological duplicates (*n* = 6) at the indicated stages of lung development. The *C*_T_ values were normalized to total RNA quantity used for the RT reaction. Data are represented as mean *C*_T_ ± SD; (**B**) *C*_T_ distribution of each putative reference gene among all mouse lung development stages analysed here. *C*_T_ values were determined as in A. For each reference gene, the mean *C*_T_ for each stage (points) and the mean *C*_T_ for all stages (line) were plotted to represent *C*_T_ variation between stages of lung development.

The ideal reference gene for a specific experiment, in which the relative quantification of gene expression should be determined by qRT-PCR, is the one whose expression is not affected by the different experimental conditions tested. To determine which one of the genes chosen for the present study is the most suitable as endogenous reference for the normalization of qRT-PCR-based expression analysis during mouse lung development, we have analysed their expression in an embryonic mouse lung at different days *post coitum* (E11.5–E18.5) and in a mouse lung at different days after birth (P1–P28) (Figure 4A). To be able to compare the qRT-PCR results obtained with the different primer pairs in the different stages of mouse lung development, we have normalized the threshold cycle (*C*_T_) with respect to the amount of RNA used in the reverse transcription reaction. The *C*_T_/μg RNA values for the genes analysed here showed a wide range from 15 to 30 depending on the stage of mouse lung development and the marker analysed. *Tuba1a* showed the most the least variability in expression among the different stages of lung development, with an average *C*_T_/μg RNA value of 19.5 and a standard deviation of 0.45 (Figure 4B). The rest of the genes showed a higher variation in their expression level during the different lung development stages. *Gapdh* and *Actb* showed moderately higher variation with average *C*_T_/µg RNA of 15.94 ± 1.25 and 16.08 ± 1.21 respectively. *Hist4h4* and *Rn18S* had an average *C*_T_/µg RNA of 20.47 ± 2.28 and 14.5 ± 3.05 and were highly unstable. Our results suggest *Tuba1a* is the most appropriate reference gene for normalizing gene expression analysis during mouse lung development.

**Figure 5:**
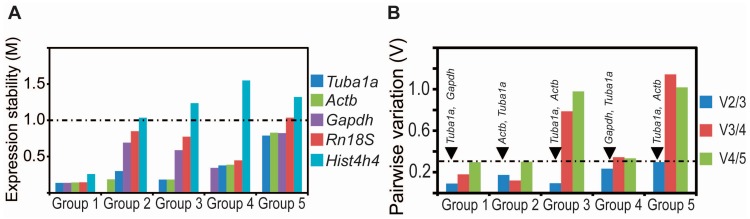
Calculation of average expression stability and pairwise variation coefficient for each of the putative reference genes. (**A**) Gene expression stability values (*M*) of the putative reference genes were calculated for each of the five experimental groups using the geNorm statistical algorithm. A lower *M* value indicated more stable gene expression. *M* values below 1.0 were considered optimal. Group 1 (branching morphogenesis, E11.5–E16.5); Group 2, (canalicular and saccular phases, E16.5–P5); Group 3 (prenatal development, E11.5–E18.5); Group 4 (postnatal development, P1–P28) and Group 5 (all stages); (**B**) The optimal number of control genes for each experimental group was determined by geNorm by calculating the pairwise variation coefficient (*V_n_*_/(*n*+1)_) between two sequential normalization factors (NF*_n_* and NF*_n_*_+1_), where n is the number of genes involved in normalization factors. Values less than 0.3 were taken as optimal. Systematic inclusion of more unstable reference genes resulted in an increase in the values. Sample grouping as in (**A**).

To further confirm this finding we have applied the statistical algorithm geNorm [20] to determine the gene expression stability (*M*) for all the putative reference genes analysed here within each of the groups of samples described before (Figure 5A and Table 3). Based on this approach, the reference genes analysed in this study were ranked in each sample group with respect to their *M* value from most stable (lowest *M* value) to least stable (highest *M* value). Keeping in mind the large heterogeneity of the samples and the vast age-related differences, a threshold for the *M* value of 1.0 was considered appropriate. For Group 1 (branching morphogenesis; E11.5–E16.5), the five tested reference genes showed M values below the threshold of 1.0 [20], indicating adequate gene expression stability to be used as internal control for normalization of qRT-PCR expression analysis for this group. Based on several reports suggesting the use of at least two different endogenous controls for the normalization of expression analysis, we calculated the normalization factors with the two most stable reference genes and systematically included additional reference genes. Pairwise variation was used to determine the optimal number of reference genes (Figure 5B, Table 4). A cut off value of 0.3 was considered optimal for the pairwise variation coefficient. For Group 1, V2/3 was 0.09, indicating that the combination of *Tuba1a* and *Gapdh* is sufficient to standardize gene expression during branching morphogenesis.

**Table 3:** Control genes ranked in order of their average expression stability. Reference genes are ranked according to the average expression stability value, *M*, as calculated by the geNorm statistical algorithm. The genes are ranked as most stable on the top. Group 1, branching morphogenesis (E11.5–E16.5); Group 2, differentiation, canalicular and saccular (E16.5–P5); Group 3, prenatal development (E11.5–E18.5); Group 4, postnatal development (P1–P28); Group 5, all stages.

Group 1		Group 2		Group 3		Group 4		Group 5	
Ranking	Stability Value (*M*)	Ranking	Stability Value (*M*)	Ranking	Stability Value (*M*)	Ranking	Stability Value (*M*)	Ranking	Stability Value (*M*)
*Tuba1a*	0.136	*Actb*	0.185	*Tuba1a*	0.182	*Gapdh*	0.343	*Tuba1a*	0.788
*Gapdh*	0.137	*Tuba1a*	0.299	*Actb*	0.183	*Tuba1a*	0.376	*Actb*	0.822
*Actb*	0.142	*Gapdh*	0.691	*Gapdh*	0.586	*Actb*	0.386	*Gapdh*	0.829
*Rn18S*	0.145	*Rn18S*	0.849	*Rn18S*	0.773	*Rn18S*	0.447	*Rn18S*	1.035
*Hist4h4*	0.258	*Hist4h4*	1.035	*Hist4h4*	1.236	*Hist4h4*	1.55	*Hist4h4*	1.321

**Table 4:** Pairwise variation coefficient calculated for sequential normalization factors for each of the five experimental groups. Pairwise variation coefficient of the most stable genes was calculated followed by sequential inclusion of less stable genes. Group 1, branching morphogenesis (E11.5–E16.5); Group 2, differentiation, canalicular and saccular (E16.5–P5); Group 3, prenatal development (E11.5–E18.5); Group 4, postnatal development (P1–P28); Group 5, all stages.

Group	V2/3	V3/4	V4/5
**Group 1**	*Tuba1a*,* Gapdh*	*Tuba1a*,* Gapdh*,* Actb*	*Tuba1a*,* Gapdh*,* Rn18S*
	0.091462	0.179303	0.296517
**Group 2**	*Tuba1a*,* Actb*	*Tuba1a*,* Gapdh*,* Actb*	*Tuba1a*,* Gapdh*,* Rn18S*
	0.173682	0.12009	0.30073
**Group 3**	*Tuba1a*,* Actb*	*Tuba1a*, *Gapdh*,* Actb*	*Tuba1a*,* Gapdh*,* Rn18S*
	0.094428	0.786888	0.978725
**Group 4**	*Tuba1a*,* Gapdh*	*Tuba1a*,* Gapdh*,* Actb*	*Tuba1a*,* Gapdh*,* Rn18S*
	0.234143	0.343936	0.333048
**Group 5**	*Tuba1a*,* Actb*	*Tuba1a*,* Gapdh*,* Actb*	*Tuba1a*,* Gapdh*,* Rn18S*
	0.29673	1.143188	1.018239

For Group 2 (cellular differentiation during canalicular and saccular phases, E16.5–P5), *Actb* and *Tuba1a* showed *M* values below the threshold of 1.0 (*M* = 0.185 and 0.299 respectively). The *M* values for *Gapdh*,* Rn18S* and *Hist4h4* were 0.691, 0.849 and 1.035 respectively, thereby showing higher variability. Further, pairwise correlation analysis showed a value of 0.17 for V2/3, which indicates that a combination of *Actb* and *Tuba1a* is sufficient to standardize gene expression during cellular differentiation.

Similar analysis was performed for all the other experimental groups. For Group 3 (Prenatal development, E11.5–E18.5),* Tuba1a* and *Actb* were the most reliable reference genes, with *M* values of 0.182 and 0.183, respectively. The pairwise correlation showed a value of 0.094 for V2/3, indicating that a combination of *Tuba1a* and* Actb* may be optimal for standardization of expression analysis in Group 3. In Group 4 (Postnatal development, P1–P28),* Gapdh*, *Tuba1a*, *Actb* and *Rn18S* had *M* values below the threshold of 1.0 (*M* = 0.343, 0.376, 0.386 and 0.447). The pairwise variation coefficient for V2/3 was 0.234, suggesting that a combination of *Gapdh* and *Tuba1a* is appropriate to use for normalization of expression analysis in Group 4. For Group 5, on including all stages of lung development, *Tuba1a*, *Actb* and* Gapdh* were the most reliable genes (*M* = 0.788, 0.822 and 0.829, respectively) and the pairwise variation coefficient for V2/3 (0.296) was marginally below the cut off value, thereby suggesting that a combination of *Tuba1a* and *Actb* may be used for normalization of expression analysis in Group 5. Our analysis further demonstrates the need to identify the most optimal reference genes for each experimental set up.

### 2.4. Expression Analysis of Two Cell Lineage Markers Using Different Normalizing Genes Suggesting Tuba1a Is the Most Suitable Endogenous Reference during Mouse Lung Development

**Figure 6:**
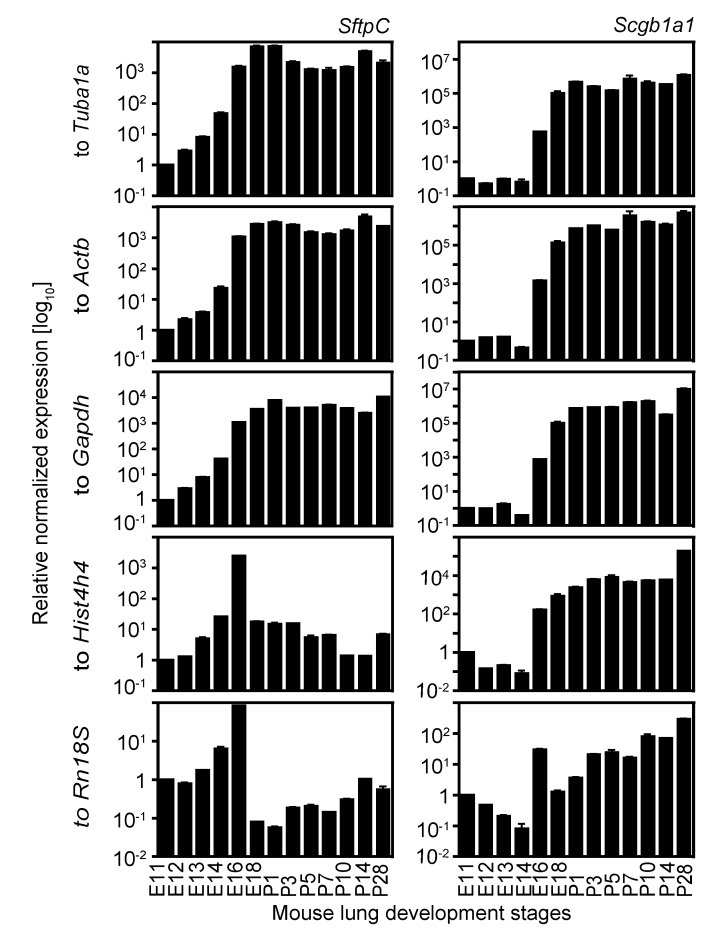
Expression analysis of two cell lineage markers during mouse lung development using different reference genes for normalization. Expression of surfactant protein C (*SftpC*, **left**) and Clara cell-specific 10 kDA protein (*Scgb1a1*, **right**) was analysed during lung development by qRT-PCR. Expression analysis in all stages of lung development was normalized to each of the five reference genes, *Tuba1a*,* Actb*,* Gapdh*, *Hist4h4*, and* Rn18S.* Days *post coitum*, *E*; postnatal, *P*. Data are represented as mean ± SD; *n* = 3.

**Figure 7:**
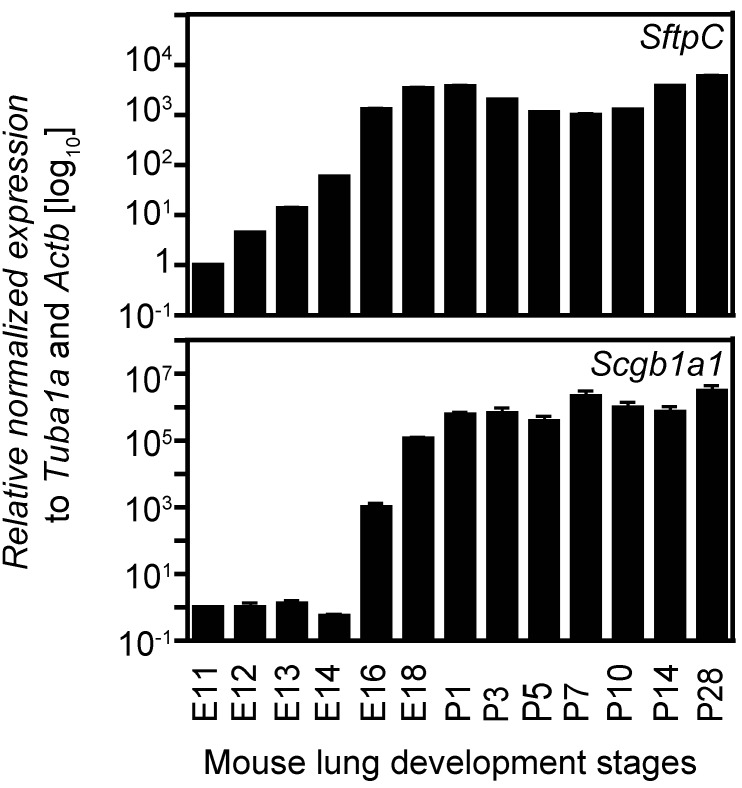
Expression analysis of two cell lineage markers during mouse lung development using a combination of two reference genes for normalization. Expression of surfactant protein C (*SftpC*) and Clara cell-specific 10 kDA protein (*Scgb1a1*) was analysed during lung development by qRT-PCR. Expression analysis in all stages of lung development was normalized to both *Tuba1a* and *Actb*. Days *post coitum*, *E*; postnatal, *P*. Data are represented as mean ± SD; *n* = 3.

From E10.5 to P5, the epithelium of the developing lung differentiates from a morphologically uniform cell population to different specialized cell types, thereby establishing a proximal-distal axis in the developing lung. The primitive lung epithelium (before E16.5) co-expresses several lineage markers including Clara cell-specific 10 kDa protein (*Scgb1a1*, also CC10) and surfactant associated protein C (*Sftpc*, also SP-C). Later in gestation (E16.5 onwards), *Scgb1a1* is a marker for the proximal epithelium, whereas *Sftpc* expression defines the distal epithelium. In the adult lung these markers are characteristic of distinct cell lineages, *Scgb1a1* of Clara cells and *Sftpc* of alveolar type II (ATII) cells. The expression of these two markers was determined by qRT-PCR at different stages of mouse lung development and normalized with respect to each of the reference genes chosen for this study (Figure 6). After normalization using *Tuba1a*, *Actb* or *Gapdh* as reference genes, the log_10_ transformed relative normalized expression of *Sftpc* and *Scgb1a1* increased from E11.5 till E16.5 and reached high levels of expression that were maintained through E18.5 to P28, consistent with previous reports [21,22,23,24,25,26]. In contrast, normalization with respect to *Hist4h4* and *Rn18S* showed an initial increase of *Sftpc* expression (till E16.5) and then a subsequent drop in expression (till P28), wherein the expression levels were similar to those in embryonic lungs (E11–E14). For *Scgb1a1*, normalization with *Rn18S* showed an initial increase of expression at E16.5 followed by a drop in expression which increased again at P3. Thus, *Hist4h4* or *Rn18S* normalization leads to altered expression profiles. These results support our previous findings (Figure 5A and Table 3) in which we have identified *Tuba1a* as the most appropriate reference gene for normalizing expression analysis during mouse lung development, followed by *Actb* or *Gapdh* depending on the phases of lung development analysed. Furthermore, log_10_ transformed relative normalized expression analysis of *Sftpc* and *Scgb1a1* using a combination of the reference genes *Tuba1a* and* Actb* (Figure 7) show even more consistent results than the standardization of the analysis using single reference genes, thereby supporting our results from the pairwise variation coefficient calculation (Figure 5B and Table 4), which suggest a combination of *Tuba1a* and *Actb* for standardization of expression analysis during lung development. The conflicting expression patterns of *SftpC* and *Scgb1a1* obtained after *Rn18S*- or *Hist4h4*-normalization as compared to the ones after normalization to *Tuba1a*,* Actb* or *Gapdh* alone or to a combination of *Tuba1a* and *Actb* highlights the importance of our analysis. Taking into consideration the complexity and the vast cellular diversity of the lung, it is crucial to validate the reference genes for every experimental setting in order to improve the consistency and to exploit the high sensitivity of qRT-PCR-based expression analysis. Inappropriate reference gene selection can result in artefacts, inaccurate interpretations and/or misleading conclusions.

## 3. Discussion

One of the most critical aspects of relative quantification of gene expression by qRT-PCR is normalization. In an ideal situation, transcript numbers are normalized and corrected after cell numbers. However, it is not always feasible to count cells. Alternatively, it is also possible to normalize with respect to the RNA quantity used for the reverse transcriptase reaction. However, both methods do not account for variations originated during RNA isolation, degradation during storage, cDNA synthesis and qPCR amplification. In order to circumvent these problems, it is recommended to normalize gene expression to an internal control, for example a “spike-in” standard or a reference gene whose expression is not affected by the conditions tested during the experiment. Due to historical reasons, the most commonly used reference genes for normalization of qRT-PCR based expression analysis include *Gapdh*,* Actb* and *Rn18S*. However, the expression of these classical reference genes has been shown to be affected by certain experimental approaches. For instance, *Gapdh* is known to be involved in additional non-glycolytic pathways and is prone to show variation in mRNA amounts [27]. It has also been reported that even though transcription rate of *Gapdh* was similar in different tissues, they contained very different amounts of mRNA [28]. *Gapdh* has also been shown to have significant differences in mRNA expression across a panel of 72 human tissue samples studied, including a maximal difference of 15-fold [29]. *Actb* has been shown to be regulated in tumour samples, as was shown in different leukaemia patient tumour samples [30]. In the case of *Rn18S*, it is widely believed that ribosomal RNA (rRNA) constitutes approximately 90% of the total RNA of the cells and it is a constant fraction of the total RNA. However, it has been shown that equal fractions of rRNA do not necessarily ensure equal fractions of mRNA [31]. Further, it has been shown that while, the sum total of 28S, 18S and 5S rRNA remains as a constant fraction of the total RNA, there are changes in the levels of each of the individual rRNA components during development [32]. In addition, it has been demonstrated that expression of rRNA genes undergoes considerable tissue specific variation, with increased expression in tissues containing a large proportion of proliferating cells (stem cells, foetal stages, lymphoid tissues, neoplastic tissues) and in tissues specialized in exocrine protein secretion (e.g., salivary glands) [33,34]. Additionally, rRNA expression increased after viral infections [35]. These observations have been explained as an increased number of ribosomes are required during cell division and protein secretion, resulting in an increased expression of rRNA genes. There are no reports of rRNA expression analysis during mouse development; however, similar observations have been made in other organisms including rats [36], zebrafish [37] and Atlantic salmon [38].Taken together, it is essential to identify suitable normalizing genes for each experimental set up.

In this study, we tested the stability of expression of five of the most commonly used reference genes during mouse lung development: *Tuba1a*,* Actb*,* Gapdh*,* Hist4h4* and *Rn18S.* Of these five putative reference genes, *Tuba1a* was found to have the least changes in expression level in all of the sample groups (Groups 1–5) analysed here, which contained different stages of mouse lung development depending on the biological process occurring at these specific stages. *Tuba1a* gene encodes a protein, alpha-tubulin, which forms a dimer with beta-tubulin to make up the microtubules which are essential for cell division and cell movement. It has been widely used as a normalization marker during mouse development [39] as well as in cancer [40]. Our results support that *Tuba1a* can be used as endogenous control for the normalization of expression analysis during branching morphogenesis (Group 1), cellular differentiation (Group 2), pre-natal and post-natal lung development (Groups 3 and 4) and general lung development (Group 5). The groups have been divided to contain increasing heterogeneity and complexity, such that Group 5 is the most variable sample. During the course of lung development, several dramatic changes take place in the embryonic lung, including proliferation and expansion of progenitor cells, specification and subsequent differentiation of the multiple cell lineages as well the formation of the capillary network. Consequently, Group 5 includes all these stages and the resulting extensive cellular diversity and the large age-related differences, indicating that it may be necessary to divide the lung development process into different experimental groups for expression analysis.

Furthermore, several reports based on different statistical algorithms suggest the use of at least two different endogenous controls for the normalization of expression analysis to increase the accuracy and reliability of the data [31]. In our study, *Tuba1a* and *Actb* were found to be the most suitable, while *Actb* and *Gapdh* were the second most suitable pair of reference genes for normalization during mouse lung development. However, due to a large number of reported pseudogenes [41], *Actb* and *Gapdh* may be used with some scepticism to support and complement the results obtained after *Tuba1a* normalization. *Hist4h4* and *Rn18S* showed the maximum variability in their expression during the different phases of mouse lung development, with a remarkable increase in their expression at later stages, supporting that they are not appropriate as internal controls for expression analysis during lung development. Our results indicate that it is essential to include validation of reference genes as an integral part of the experiment design. Inappropriate reference genes may have a profound influence on the results which includes divergent outcomes leading to inaccurate data interpretation.

## 4. Experimental Section

### 4.1. Ethics Statement

Mouse work was performed in compliance with the German Law for Welfare of Laboratory Animals. The permission to perform the experiments presented in this study was obtained from the Regional Council (Regierungspräsidium in Darmstadt, Germany). The numbers of the permissions are IVMr46-53r30.03.MPP04.12.02 and IVMr46-53r30.03.MPP06.12.01. Animals were killed for scientific purposes according to the law mentioned above which comply with national and international regulations. Thus, trained individuals performed all the animal work and took precautions to reduce animal suffering. As method of sacrifice, adult animals were anaesthetized using 5% isoflurane followed by cervical dislocation. Death was confirmed by respiratory arrest and stop of heartbeat. Embryos were then isolated from the mice and kept for short time in ice cold PBS. Prior to organ micro dissection, the embryos were killed by decapitation using very sharp scalpels. Similarly, for organ isolation from postnatal mice (P1–P14), the animals were anaesthetized using 3% isoflurane followed by decapitation before organ dissection.

### 4.2. Work with Animals

Animals were housed under controlled temperature and lighting (12/12-h light/dark cycle), fed with commercial animal feed and water *ad libitum*. Time pregnant C57Bl6 wild type mice were sacrificed on post coitum days 11.5, 12.5, 13.5, 14.5, 16.5 and 18.5 (E11–18, day of plug = E0.5) according to standard methods described above and embryos were collected. Staging was confirmed by somite numbers till E14.5, and subsequently, mouse embryos were staged morphologically. Embryonic lungs were isolated using microscopy based manual dissection using previously established protocols [42] and flash frozen in liquid nitrogen. C57Bl6 pups were sacrificed on day 1, 3, 5, 7, 10, 14 and 28 (P1–28, day of birth = P0) and their lungs were also harvested after perfusion with PBS and flash frozen in liquid nitrogen.

### 4.3. RNA Isolation, cDNA Synthesis and qRT-PCR

In order to have sufficient material for RNA isolation, five lungs were used for the embryonic stages, while for post natal stages, three lungs were used. Total RNA was isolated from the lungs for using the RNeasy plus mini kit (Qiagen, Hilden, Germany), including an on-column RNase-free DNase digestion. RNA integrity was examined using agarose gel electrophoresis. RNA was quantified and purity was verified using the Nano-Drop 2000c UV–vis spectrophotometer (Thermo Fisher Scientific, Waltham, MA, USA) and 1 µg of the total RNA was used for cDNA conversion using the High Capacity cDNA Reverse Transcription kit (Applied Biosystems) using random hexamers following manufacturer’s instructions. cDNA samples were diluted 1:4 and 1 µL of each was used in a 10 µL qRT-PCR reaction using the SYBR^®^ Green PCR Master mix (Applied Biosystems, Foster City, CA, USA) and 250 nM each forward and reverse primers. Transcript levels were analysed on the ABI Step One Plus PCR machine over 40 cycles of 95 °C for 15 s and 60 °C for 1 min in a two-step thermal cycle preceded by a 10 min denaturation at 95 °C. All reactions were performed in triplicate and analysis was performed using five biological replicates. Primers were designed for expression analysis for each of the genes using Geneious (Geneious version 6.1 created by Biomatters [43]). Primer characteristics, including annealing temperature, formation of primer dimers and secondary structures were checked using the jPCR [15] and the Sigma Aldrich oligo Evaluator online tool [44]. In addition, specificity of the primers was checked using the NCBI primer BLAST tool [45]. Absence of genomic DNA was confirmed by −RT reactions for all RNA pools using gene specific primers*.*

For the analysis of gene expression, relative amounts of both genes of interest were calculated normalized to each reference gene based on the formula, relative expression = *E*^Δ*C*T^, where *E* represents the amplification efficiency (*E*) for each gene and Δ*C*_T_ is the difference in the *C*_T_ from each target sample and calibrator (Δ*C*_T_ = *C*_T_(calibrator) − *C*_T_(target)). The normalization factor, calculated using the geometric mean of the relative expression of the two reference genes selected for normalization, was used to obtain the relative normalized expression based on two reference genes simultaneously [46,47].

### 4.4. Sequencing of PCR Products

PCR products were gel purified after qRT-PCR and cloned into pJET1.2 blunt cloning vector using the CloneJET PCR cloning kit (Thermo Scientific, Waltham, MA, USA). Five clones for each gene were selected and sent for sequencing after plasmid purification. Sequence analysis was performed using Geneious (Geneious version 6.1 created by Biomatters [43]).

### 4.5. Stability Ranking of Candidate Reference Genes

For the calculation of the stability of the reference genes, the samples analysed here were combined into five experimental groups: branching morphogenesis (Group 1; E11.5–E16.5 including late embryonic and pseudoglandular phases), cellular differentiation (Group 2; E16.5–P5 including canalicular and saccular phases), pre-natal lung development (Group 3; E11.5–E18.5 including embryonic, pseudoglandular, canalicular and initial saccular phases); post-natal lung development (Group 4; P1–P28 including late saccular and alveolar phases) and general lung development (Group 5; E11.5–P28 including all phases of lung development). Reference gene validation was performed using the statistical algorithm geNorm [20]. geNorm uses a pair-wise approach, assuming that stable reference genes would have similar intergroup variation, leading to small pair wise differences. Stability is estimated by the average pair wise variation of the given reference gene with all the other genes in the validation set. Reference genes are then ranked through successive step wise exclusion of the least stable gene. Normalization factors were calculated by geometric averaging of the reference genes in a group. Subsequently, pairwise Variation *V_n_*_/(*n*+1)_ was estimated by including stepwise reference genes until the (*n* + 1)th gene had no significant contribution to the stability of expression.

## 5. Conclusions

It is necessary to select an appropriate reference gene (ideally two) for each experimental setup as an internal control for the normalization of relative expression analysis. The ideal reference gene for a specific experiment is the one whose expression is not affected by the different experimental conditions tested. This analysis demonstrates that *Tuba1a* is the most suitable endogenous reference for the normalization of qRT-PCR-based expression analysis during the different phases of lung development.
